# Metropolitan Radical Federation and the Funds

**Published:** 1911-04-22

**Authors:** Edward Ganity

**Affiliations:** Hon. Secretary. 37 Hall Street, City Road, E.C.


### Editor's Letter-Box.

### METROPOLITAN RADICAL FEDERATION AND THE FUNDS.

*To the Editor of* The Hospital.

I am directed by the Metropolitan Radical Federation publicly to express the gratification of all its members at the rebuke administered by King Edward's Hospital Fund, in its recently published report, to the managers of St. George's Hospital for diverting unjustified sums of money from the coffers of the hospital to those of the school.

We shall observe with care whether the managers of the hospital and school obey the directions of King Edward's Fund, to refund to the hospital monies thus taken from the services of the sick poor, or whether the authorities of both hospital and school aggravate their contumelious conduct in the past by defiance of the King's Fund in the present and future.

Inasmuch as the Hospital Sunday Fund, under the presidency of the Lord Mayor, has not protected the subscriptions given in places of worship from being granted to St. George's Hospital, although the conduct of the authorities of that hospital has suffered condemnation at the hands of the King's Fund, the Metropolitan Radical Federation endorse the suggestion made by Mr. Stephen Coleridge in the *Times* of April 3, that in future the entire Mansion House Fund should be handed over annually by the Lord Mayor to King Edward's Fund for distribution by it.

But the Federation entertains the earnest hope that in any such concentration of the two funds an adequate representation of the working classes, for whose benefit the whole of both the funds is subscribed, should be provided for on the Committee of the King's Fund.

Your obedient servant,
Edward Ganity, Hon. Secretary.

37 Hall Street, City Road, E.C.
April 11, 1911.

[*** It would be interesting to learn on what grounds this claim for the participants in charitable relief to manage its distribution is held to be justifiable.—Ed. The Hospital.]

